# Changes in functional connectivity in people with HIV switching antiretroviral therapy

**DOI:** 10.1007/s13365-020-00853-0

**Published:** 2020-06-04

**Authors:** Sofia Toniolo, Mara Cercignani, Borja Mora-Peris, Jonathan Underwood, Jasmini Alagaratnam, Marco Bozzali, Marta Boffito, Mark Nelson, Alan Winston, Jaime H. Vera

**Affiliations:** 1 Clinical Imaging Sciences Centre, Brighton and Sussex Medical School, Knightsgate Road, Falmer Campus, Brighton, BN1 9RR UK; 2 Present Address: Nuffield Department of Clinical Neurosciences, University of Oxford, New Radcliffe House, Walton St., Oxford, OX2 6BW UK; 3 Department of Infectious Disease, Faculty of Medicine, St Mary’s Campus, Imperial College London, Praed Street, London, W2 1NY UK; 4 Division of Infection and Immunity, School of Medicine, Cardiff University, UHW Main Building, Heath Park, Cardiff, CF14 4XN UK; 5 Department of HIV Medicine, Chelsea and Westminster NHS Foundation Trust, 369 Fulham Road, London, SW10 9NH UK; 6 Department of Global Health and Infection, Brighton and Sussex Medical School, Brighton, BN1 9PX UK

**Keywords:** HIV, fMRI, Neuropsychological assessment, Memory, Attention

## Abstract

We assessed changes in functional connectivity by fMRI (functional magnetic resonance imaging) and cognitive measures in otherwise neurologically asymptomatic people with HIV (PWH) switching combination antiretroviral therapy (cART). In a prospective study (baseline and follow-up after at least 4 months), virologically suppressed PWH switched non-nuclease reverse-transcriptase inhibitors (NNRTI; tenofovir-DF/emtricitabine with efavirenz to rilpivirine) and integrase-strand-transfer inhibitors (INSTI; tenofovir-DF/emtricitabine with raltegravir to dolutegravir). PWH were assessed by resting-state fMRI and stop-signal reaction time (SSRT) task fMRI as well as with a cognitive battery (CogState™) at baseline and follow-up. Switching from efavirenz to rilpivirine (*n* = 10) was associated with increased functional connectivity in the dorsal attention network (DAN) and a reduction in SSRTs (*p* = 0.025) that positively correlated with the time previously on efavirenz (mean = 4.8 years, *p* = 0.02). Switching from raltegravir to dolutegravir (*n* = 12) was associated with increased connectivity in the left DAN and bilateral sensory-motor and associative visual networks. In the NNRTI study, significant improvements in the cognitive domains of executive function, working memory and speed of visual processing were observed, whereas no significant changes in cognitive function were observed in the INSTI study. Changes in fMRI are evident in PWH without perceived neuropsychiatric complaints switching cART. fMRI may be a useful tool in assisting to elucidate the underlying pathogenic mechanisms of cART-related neuropsychiatric effects.

## Introduction

Earlier treatment initiation and wider access to combination antiretroviral therapy (cART) have resulted in a significant decline in the incidence of HIV-associated central nervous system (CNS) diseases (Garvey et al. 2011). Despite this, neuropsychiatric events, such as cognitive deficits, anxiety, depression and insomnia, are frequently reported in people with HIV (PWH) on effective cART (Knights et al. 2017). Alongside traditional pathogenic mechanisms such as the legacy effect of HIV in CNS prior to cART initiation and CNS immune activation, antiretroviral toxicity may be a potential pathogenic factor responsible for the occurrence of these neuropsychiatric symptoms (Underwood et al. 2014). Indeed, efavirenz, a highly efficacious non-nucleoside reverse-transcriptase inhibitor (NNRTI), has been associated with neuropsychiatric symptoms in 20–40% of patients which may persist for several years after treatment initiation (Zhuang et al. 2017; Sacktor et al. 2006; McCutchan et al. 2007). Integrase-strand-transfer inhibitors (INSTI) have also been linked to an increased risk of neuropsychiatric adverse events, with dolutegravir being associated with the highest rate of discontinuation compared with other INSTI (2–6%) (Elzi et al. 2017; Hoffmann et al. 2017). What impact antiretroviral drugs might have on brain activity and how it correlates with covert neuropsychiatric symptoms remains poorly described.

Functional MRI (fMRI) is an imaging modality that may provide valuable information on the effects of different cART regimens on the CNS by measuring changes in brain functional connectivity at rest or in response to tasks (Wise and Tracey 2006). The aim of this study is to assess changes in functional connectivity by fMRI and cognitive and behavioural measures in neurologically asymptomatic PWH participating in studies assessing changes in NNRTI and INSTI.

## Methods

### Subject selection and study design

PWH were enrolled in two separate phase IV, open-label studies conducted in 3 UK sites (Chelsea and Westminster Hospital, St. Mary’s Hospital London and the Royal Sussex County Hospital) between January 2016 and January 2018.

**Fig. 1:**
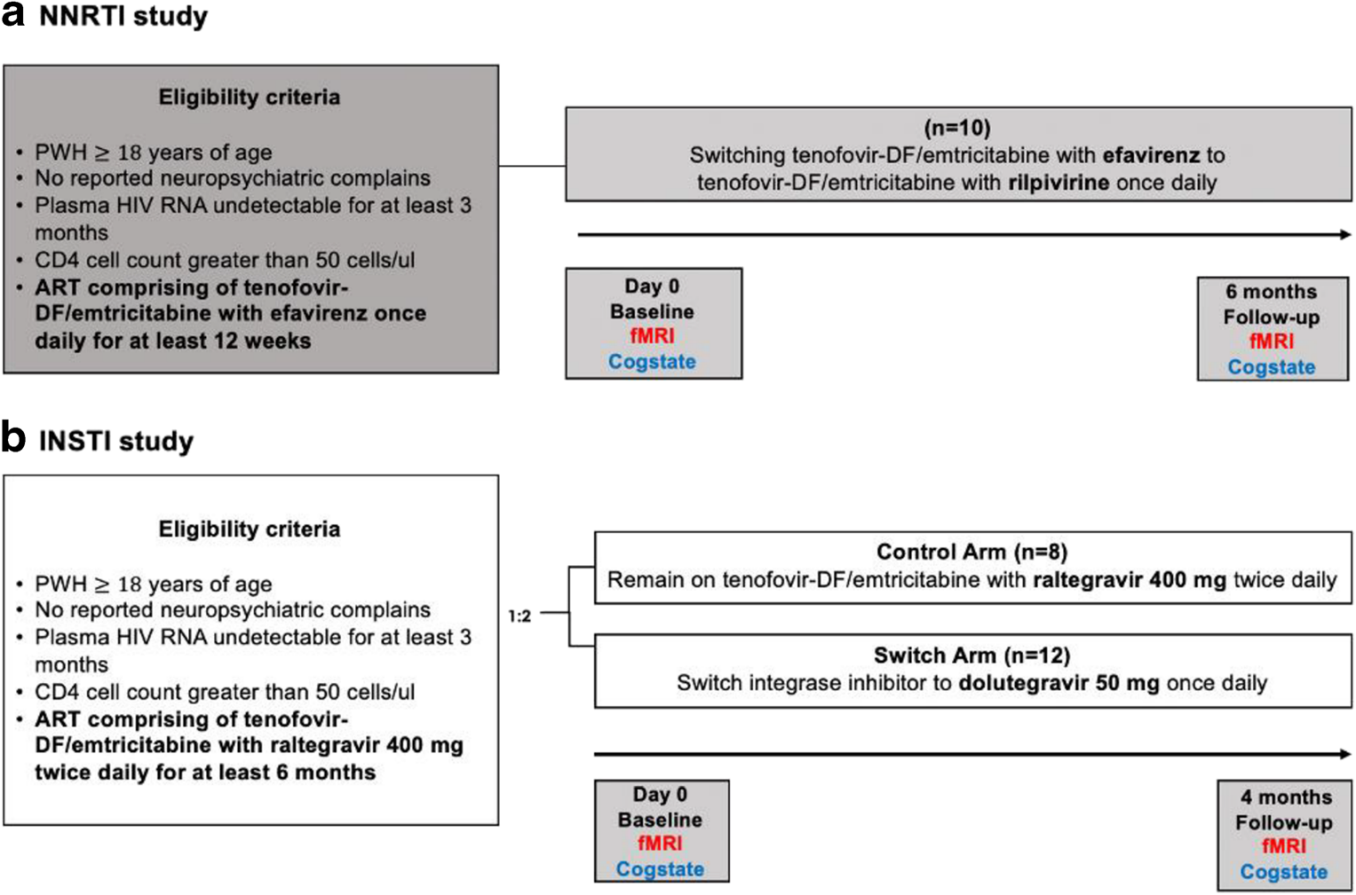
Study design for the NNRTI and INSTI studies

Inclusion criteria for both studies included no history of subjective or objective neurological or psychiatric symptoms, age ≥ 18 years, plasma HIV RNA < 40 copies/mL for at least 3 months, CD4 cell count > 50 cells/μL and being stable on the same cART regimen for at least 12 weeks (tenofovir-DF/emtricitabine/efavirenz for the first study (NNRTI study) and tenofovir-DF/emtricitabine and raltegravir for the second study (INSTI study) (see Fig. 1).

Subjects were excluded if they had previous exposure to rilpivirine in the NNRTI study and dolutegravir in the INSTI study, viral hepatitis co-infection, evidence of HIV drug resistance mutations, use of disallowed concomitant medication or current self-reported recreational drug use or alcohol abuse.

In the NNRTI study, all eligible participants (*n* = 10) were switched from the fixed-dose combination tenofovir-DF/emtricitabine/efavirenz to tenofovir-DF/emtricitabine/rilpivirine for 6 months. In the INSTI study, participants were randomized in a 1:2 fashion to either remain on the same cART drug regimen or switch to tenofovir-DF/emtricitabine and dolutegravir 50 mg once daily for the duration of the study period (4 months). Randomization accounted for a total of 12 patients switching to dolutegravir and of 8 patients that remained on raltegravir. At each study visit, all study participants were specifically questioned and assessed by a research nurse or doctor about any psychiatric or neurological adverse events.

### Standard protocol approvals, registrations and patient consents

The studies were conducted in accordance with the Declaration of Helsinki, the Uniform Requirements for Manuscripts submitted to Biomedical Journals, were approved by the research ethics committee, the UK regulatory authority and Medicines and Healthcare products Regulatory Agency (MRHA) and were registered with the EudraCT trials database (2014-002284-15) and (2014-003710-84). All subjects gave written informed consent prior to study initiation for both studies. The contracts between academic institutions and the funders stated clearly independence in terms of design, analysis and dissemination of the results. The present manuscript was analysed by investigators that have never received any funding from HIV-related pharmaceutical companies as described in the disclosures. Furthermore, the neuroimaging analysis was conducted blinded to the drug names to prevent the imaging specialist from bias.

### Study procedures

#### Clinical assessments

##### Cognitive testing

Cognitive testing was performed at baseline and at the final study visit using CogState™ (CogState™ Ltd., Melbourne, Australia), a computerized battery previously validated in PWH (Cysique et al. 2006) to assess processing speed, attention, visual learning, spatial problem solving, working memory and executive functions.

Cognitive results were compared with standardized normative data provided by CogState™ and transformed into *Z*-scores. Within the study time point, changes were tested using the Wilcoxon signed-rank test.

##### Patient-reported outcome measures

Participants completed the following questionnaires at baseline and follow-up: Patient Health Questionnaire*-*9 (PHQ-9) (Kroenke et al. 2001), Beck’s Depression Inventory (BDI) (Beck et al. 1961), Pittsburgh Sleep Quality Index (PSQI) (Buysse et al. 1989) and Hospital and Anxiety and Depression Scale (HADS) (Zigmond and Snaith 1983). Within the study time point, changes were tested using the Wilcoxon signed-rank test.

#### Imaging assessments

##### Resting-state network fMRI analysis

*Data pre-processing*: All imaging was obtained using a 3.0T MR scanner (SIEMENS MAGNETOM Verio syngo MR B17) with a 32-channel head coil for both visits. The following scans were collected from each studied subject at baseline and follow-up: a high-resolution 3D fluid-attenuated inversion recovery (FLAIR) sequence (TE = 394 ms, TR = 5000 ms, TI = 1800 ms, field-of-view = 250 mm, 160 contiguous slices of 1 mm thickness, voxel size = 1 mm3); a three-dimension (3D) magnetization-prepared rapid gradient-echo (MPRAGE) (TE = 2.98 ms, TR = 2300 ms, TI = 900 ms, flip angle = 9°, field-of-view = 256 mm, 160 contiguous slices of 1 mm thickness, voxel size = 1 mm3); and a BOLD response-sensitive echo-planar imaging (EPI) sequence (TE = 30 ms, TR = 2000 ms, flip angle = 80°, field-of-view = 192 mm, 35 interleaved slices of 3 mm thickness with no gap, voxel size = 3 mm3, duration 10.06 min) for resting-state fMRI. Participants were instructed to remain still with their eyes closed and not think of anything specific while trying to stay awake. Nine out of 10 in the NNRTI study, 9 out of 11 people on the dolutegravir arm and 7 out of 8 on the raltegravir arm in the INSTI study gave consent to perform the fMRI task so an additional sequence with the same parameters as the resting state was acquired while participants performed the stop-signal task (SST) behavioural task. According to the inclusion criteria, FLAIR scans were reviewed to exclude the presence of significant macroscopic brain abnormalities. For all MRI scans, resting-state fMRI data analysis was carried out using FMRIB Software Library (FSL) (Jenkinson et al. 2012). We first discarded the first 4 volumes to allow steady-state magnetization. Each dataset underwent motion correction with MCFLIRT (Jenkinson et al. 2002a), non-brain tissue removal with brain extraction tool (BET) (Jenkinson et al. 2002b), nonlinear registration of the functional images to the main structural image with FNIRT (FMRIB’s nonlinear image registration tool) and denoising using independent component analysis-based Automatic Removal Of Motion Artifacts (ICA-AROMA) (Pruim et al. 2015). The images extracted from AROMA were then normalized to the Montreal Neurological Institute (MNI) standard space (2 mm isotropic). Grand-mean intensity normalization and spatial smoothing with a Gaussian kernel of 5.0 mm full width at half-maximum (FWHM) were subsequently performed.

*fMRI RSN first-level analysis:* A group-level ICA was performed across all subjects and all scans using MELODIC (Beckmann and Smith 2004). First, the previously pre-processed 4D dataset was temporally transformed by concatenation into a single time series. This new 4D image was then separated into different independent components (ICs).

We decided to use 2 different IC dimensionalities (number of IC components) for maximizing the matching between the data generated by our sample and previously described RSNs. In the NNRTI study, we did not a priori set the number of component, and within the 38 group-level IC maps originated by MELODIC, we selected 8 cognitively relevant networks, i.e. default mode network (DMN), executive network (EXE), dorsal attention network (DAN), ventral attention network (VAN), anterior limbic network (ANTLIMB), posterior limbic network (POSTLIMB) and left and right frontoparietal network (LFP and RFP).

For the INSTI study, we set the ICs to 20 according to the 20 RSN model by Smith et al. (Smith et al. 2009), and within the group-level IC maps originated by MELODIC, we selected 10 networks of interest, i.e. DMN, EXE, salience network (SAL), DAN, VAN, limbic network (LIMB), LFP and RFP, sensory-motor network (SM) and associative visual network (VISAS). The identification of each network was defined according to previously described RSNs (Smith et al. 2009; Thomas Yeo et al. 2011; Dipasquale and Cercignani 2016; Laird et al. 2011), with the associative visual network corresponding to the lateral visual areas by Smith et al. (Smith et al. 2009). A visual inspection of each component spatial profile to ensure the effectiveness of the matching between the RSNs originated by MELODIC and previously described RSNs was performed. This procedure allows us to check which RSN model describes best the networks generated by our data sample.

*fMRI RSN second-level analysis:* Subject-specific IC maps were obtained using dual regression (Filippini et al. 2009) and, subsequently, a second-level analysis testing the between-session group difference was performed using FSL randomize (Winkler et al. 2014) through a 2-sample *t* test on the between-session difference image with the number of iterations set to 2000. A binary mask of every network was obtained by a one-sample *t* test and the analysis was restricted within the mask. We estimated 4 contrasts: within-session means and the within-subject, between-session differences. The second-level inference was thresholded at a family-wise error (FWE)-corrected probability of *p* < 0.05 with threshold-free cluster enhancement (TFCE).

##### Stop-signal task fMRI analysis

**Fig. 2:**
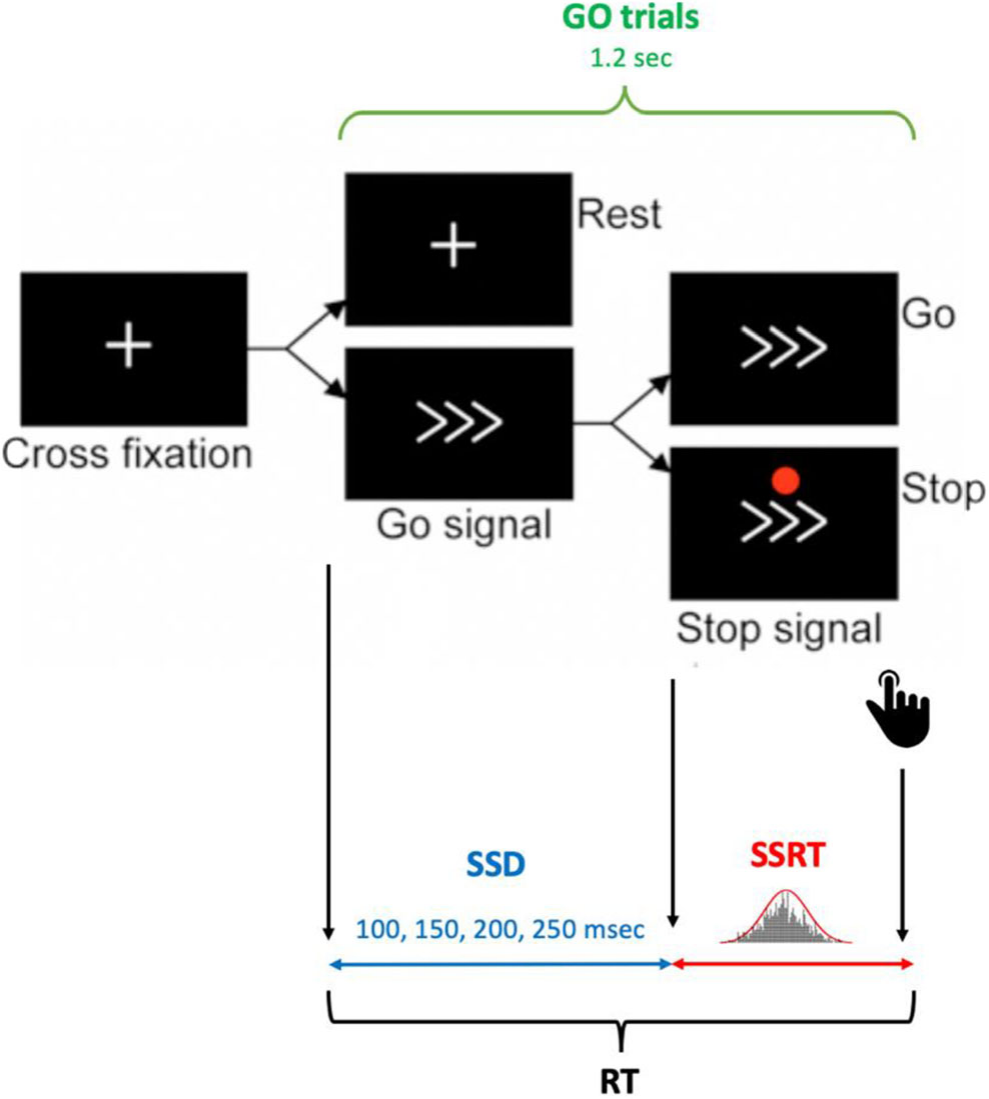
Behavioural task (SST) experimental model. SSD, stop-signal delay; RT, response time; SSRT, stop-signal response time

Subjects performed a response inhibition task, the stop-signal task (SST) inside the scanner as task fMRI at baseline and follow-up (see Fig. 2). The SST measures the efficiency of response inhibition, with lower SSRTs (stop-signal response times) indicating less time required to inhibit a response (Rae et al. 2014).

Briefly, participants performed a speeded response task (e.g. indicating whether an arrow points to the left or right). On “GO” trials, an arrow pointing to the left or right was presented, and the subjects were instructed to respond as quickly as possible. On “STOP” trials, a stop signal was presented concurrently with or just after the target, and participants had to withhold their response. The onset time of the stop signal relative to the go stimulus or stop-signal delay (SSD) changed dynamically throughout the experiment and the efficiency of response inhibition was estimated by observing the effects of varying the SSD on the SSRT.

SSD changed dynamically throughout the experiment depending on the subject’s performance, increasing by 50 ms if the subject inhibited successfully on a STOP trial or conversely decreasing the SSD by 50 ms if inhibition was unsuccessful. Four step-up and step-down algorithms (staircases), with initial SSD values of 100, 150, 200 and 250 ms respectively, were used to ensure convergence to inhibition of 50% trials by the end of the experiment. Average SSD was computed, for each subject, using the values of the four staircases after the subject had converged on 50% inhibition (Aron and Poldrack 2006), while SSRT was estimated by subtracting average SSD from median correct RT in the GO trials. To improve subject attentive engagement and motivation over time, a “negative feedback” cue was presented based on whether the last reaction time (RT) was over 0.95% of the previous RTs. SSRT, RT and accuracy were compared between time points. Firstly, we used the Shapiro-Wilk test to assess normality distribution and then either a paired *t* test or a Mann-Whitney *U* test according to the normality of the data.

We then looked at associations between SSRT, RT and accuracy and clinical parameters. As most of the participants in both studies were of the male gender, no associations between gender and SSRT/RT were investigated. No significant associations between SSRT/RT parameters and age were observed. We selected 5 covariates of interest (years with HIV, years on cART, CD4 count, CD4/8 ratio and years on the drug) and we performed an ANOVA with repeated measures on SSRTs, RTs and accuracy using these specific clinical data as covariates.

*Data pre-processing and fMRI SST first-level analysis:* All image processing was done in Statistical Parametric Mapping Version 12 (SPM12). The first 4 images were discarded in order to allow steady-state magnetization. Images were realigned to the mean image, slice time corrected, normalized to the MNI standard template and smoothed with a Gaussian kernel of 5.0 mm FWHM.

The following contrasts were estimated and taken to the second level for each session: STOP correct trials > GO trials and GO trials > STOP correct trials.

*fMRI SST second-level analysis:* For the NNRTI study, we performed a paired *t* test between images at baseline and after 6 months. For the INSTI study, the between-session difference image was assessed using ImCalc SPM function in order to obtain the final within-subjects’ image and a two-sample *t* test was used to assess the between-group difference in the 2 groups.

For all results, we examined two different contrasts, respectively assessing whether participants showed increased or decreased fMRI activation between sessions (NNRTI study) or between groups (INSTI study) for both conditions: STOP > GO trials and GO > STOP trials. The resulting statistic images were thresholded at *p* < 0.05 cluster-based FWE with cluster formed using *p* < 0.001 at the voxel level.

##### Data availability statement

The full anonymized dataset for this project will be deposited in the University of Sussex research repository as part of the University’s Research Data Management Policy where all data for this study can be accessed upon request and approval by the research ethics committee.

## Results

Ten participants from the NNRTI study and 20 participants from the INSTI study (8 in the control arm and 12 in the switch arm) completed study procedures. Baseline characteristics are presented in Table 1.

**Table 1:** Demographic characteristics for NNRTI and INSTI studies

Parameter	NNRTI study (efavirenz to rilpivirine)	INSTI study (raltegravir to dolutegravir)	
		Control arm	Switch arm
Number	10	8	12
Age, years median (IQR)	52 (14)	39.5 (15.5)	43 (13)
Male, *n* (%)	10 (100)	7 (87.5)	12 (100)
White ethnicity, *n* (%)	10 (100)	5 (62.5)	9 (69.2)
CD4+ count at study entry (cells/μL), median (IQR)	600 (256)	688 (395)	736 (237)
HIV RNA viral load < 40 copies/mL, *n* (%)	10 (100)	8 (100)	12 (100)

Demographic characteristics for NNRTI and INSTI studies

One MRI scan in the switch arm of the INSTI study at follow-up was corrupted by severe artefacts, so the subject was excluded from the group MRI analysis. Study drugs were well tolerated with all patients reporting over 95% adherence to therapy. No safety or laboratory concerns related to any of the study drugs were observed. At 4 months, plasma HIV RNA was < 40 copies/mL in all subjects for both studies.

### Effect of switching cART on cognitive testing

In the NNRTI study, no statistically significant differences between the time points were found in global cognitive scores; however, a significant improvement in cognitive tests assessing executive functions, working memory and visual-motor control were observed (Groton Maze Learning Test, One Back Test and Groton Maze Chase Test) (see Table 2). No changes in individual cognitive tests were observed in the INSTI study.

**Table 2:** Changes in cognitive function by study arm (NNRTI study)

			Baseline	6 months	Changes at 6 months from baseline	*p* value
Cognitive test	Cognitive domain	Outcome measure	*Z*-scores (SD)	*Z*-scores (SD)	Mean score difference (95% CI)	
Identification	Attention	Speed of performance log10 milliseconds	− 0.39 (0.48)	− 0.34 (0.81)	0.05 (− 0.80/0.91)	0.88
Detection	Psychomotor function	Speed of performance log10 milliseconds	− 0.65 (0.93)	− 0.72 (0.64)	− 0.07 (− 0.5/0.42)	0.75
One Card Learning	Visual learning	Accuracy of performance	− 0.66 (1.09)	− 0.62 (1.6)	0.03 (− 1.11/1.18)	0.94
One Back	Working memory	Speed of performance log10 milliseconds	− 0.01 (0.97)	− 2.13 (1.02)	− 2.11(− 2.7/− 1.4)	0.001
Groton Maze Chase	Visual-motor control	Moves per second	0.10 (1.02)	− 0.61 (0.9)	− 0.72 (− 1.20/− 0.20)	0.011
Groton Maze Learning	Executive function	Total number of errors	− 0.06 (0.98)	− 2.13 (1.2)	− 2.06 (− 2.90/− 1.22)	0.003
SET shifting	Executive function	Total number of errors	− 0.04 (0.96)	0.03 (1.02)	0.07 (− 0.64/0.80)	0.81
Global cognitive *Z*-score	Global cognition	*Z*-score	− 0.19 (24)	− 0.58 (0.78)	− 0.24 (− 1.05/0.5)	0.24

*SD*, standard deviation; *CI*, confidence interval; *p* value, two-tailed. Lower scores at Identification, Detection, One Back, Groton Maze Chase, Groton Maze Learning, SET shifting, Global cognitive score indicate better performance. Higher scores at the One Card Learning task indicate better performance

### Effect of switching cART on PROMs

In the NNRTI study, a 3% improvement in sleep quality (median change (IQR) from baseline: 3 (− 2 to 10); *p* = 0.009 for the PSQI) and a 4.8% improvement in anxiety symptoms (median change (IQR) from baseline: 4.8 (− 4.8 to 33); *p* = 0.053 for HADS) at follow-up were observed following the switch. No differences in changes of other PROMs were observed and no statistically significant differences were observed in the INSTI study.

### Effect of switching cART on resting-state networks fMRI

**Fig. 3:**
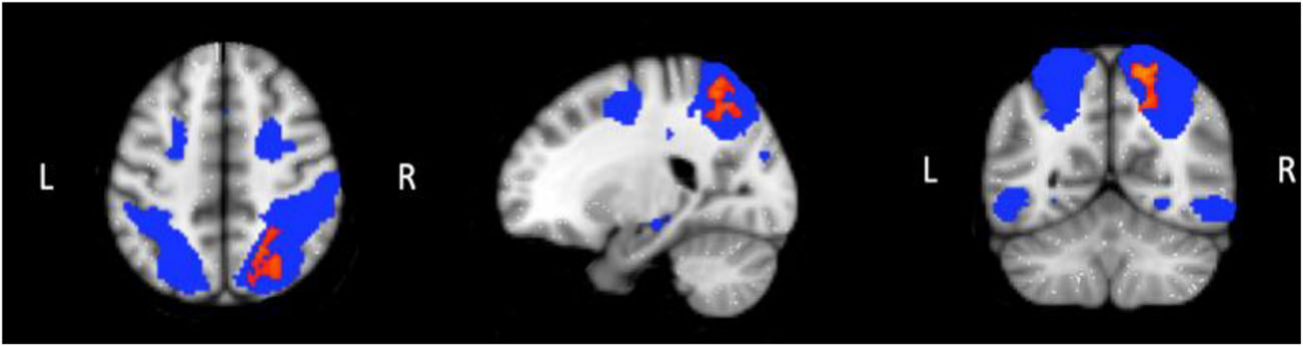
Resting-state connectivity after switching to rilpivirine compared with the baseline. Red: areas of enhanced connectivity within the DAN (right superior parietal lobule). Blue: DAN mask. L, left; R, right

In the NNRTI study, over the study period, we observed significantly enhanced connectivity of the dorsal attention network (DAN), and in particular of the right superior parietal lobule (see Fig. 3). No other changes in functional connectivity among the other 7 networks examined were observed.

**Fig. 4:**
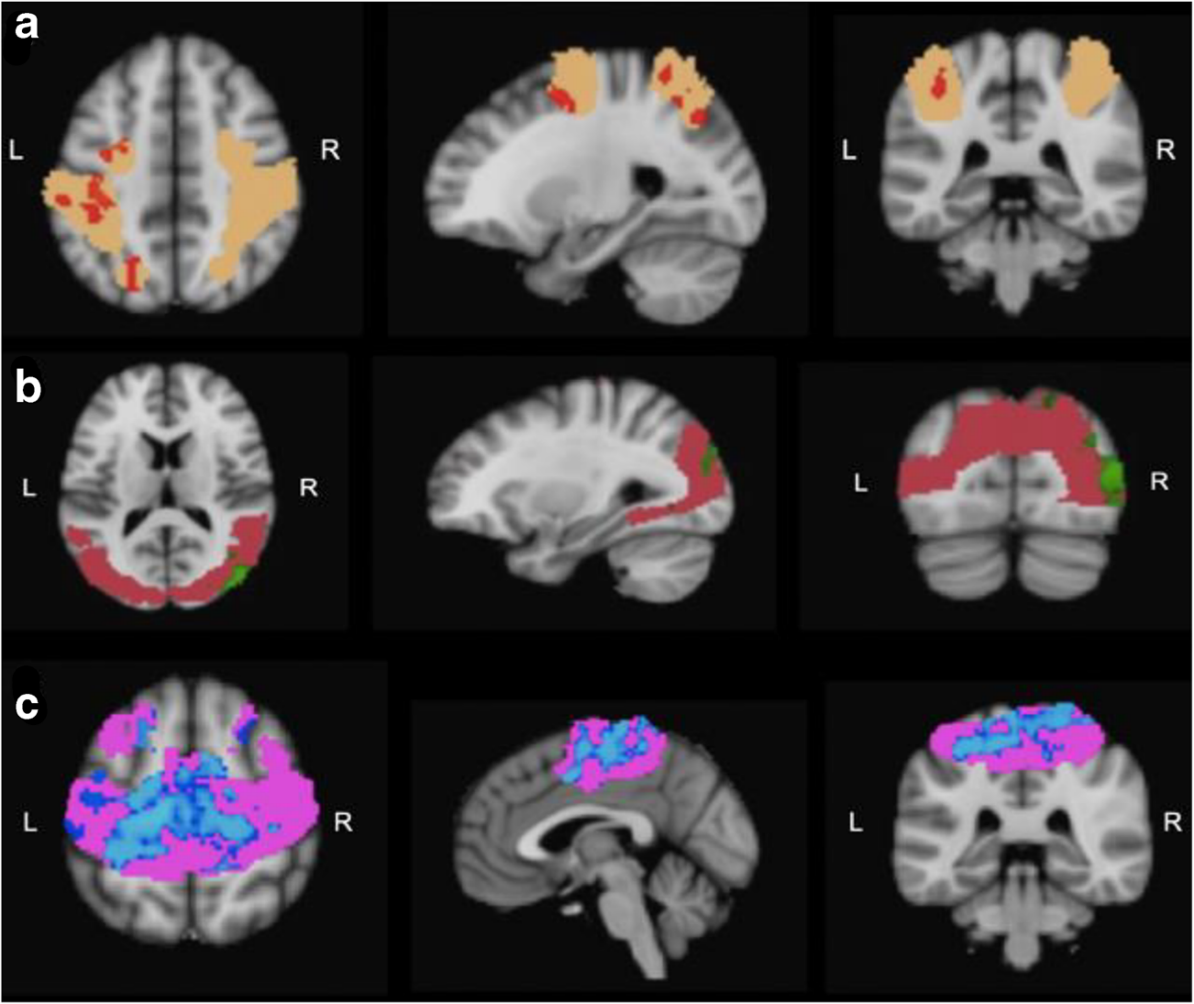
Resting-state connectivity after switching to dolutegravir compared with the baseline. **a** DAN: red, areas of enhanced connectivity within the DAN (left middle frontal gyrus, left superior parietal lobule, left supramarginal gyrus and the left superior frontal gyrus). Orange, DAN mask. **b** VISAS: green, areas of enhanced connectivity within the VISAS network (right lateral occipital cortex). Magenta, VISAS mask. **c** SM: blue, areas within the SM network (superior and medial frontal gyrus and precentral gyrus) of enhanced connectivity. Pink, SM mask. L, left; R, right

In the INSTI study, significantly increased connectivity in the DAN, VISAS (associative visual) and SM (sensory-motor) networks was observed over the study period (see Fig. 4). Within the DAN, the brain areas that showed an increase in functional connectivity were the left middle frontal gyrus (within the frontal eye fields), left superior parietal lobule, left supramarginal gyrus and left superior frontal gyrus. Regarding the VISAS, we found an enhanced connectivity within the right lateral occipital cortex. In the SM network, we found a wider increase of functional connectivity bilaterally in the superior and medial frontal gyrus and precentral gyrus.

### Effect of switching cART on behavioural measures (SST)

In the NNRTI study, a reduction in SSRT was observed after switching from efavirenz to rilpivirine (*p* = 0.025). Participants showed good response accuracy in both conditions, thus confirming good compliance to the task. The reduction in SSRTs between the two conditions was positively correlated with the duration of efavirenz exposure, *F*(1,3) = 20.976, *p* = 0.020, *η*^2^ = 0.875. No statistical correlation between the other values and changes in SSRTs was observed (see Table 3). In the INSTI study, SSRTs (*p* = 0.606), mean RTs (*p* = 0.724) and accuracy (*p* = 0.252) were not significantly different between the two groups or between time points.

**Table 3:** Association between clinical data and behavioural results for the NNRTI study

	Mean values (SD)	SSRT (*p*)	RT (*p*)	Accuracy (*p*)
Years with HIV	14.0 (5.2)	0.99	0.645	0.59
Years on efavirenz	4.8 (3.1)	0.02	0.18	0.20
CD4 count (cells/μL)	601.7 (168.7)	0.38	0.35	0.56
CD8 count (cells/μL)	551.6 (150.8)	0.89	0.35	0.56
CD4/CD8 ratio	1.1 (0.3)	0.51	0.35	0.69

Years with HIV and years on efavirenz are expressed in years, CD4 and CD8 count in (cells/μL). (*p*), *p* value, obtained using a repeated-measure ANOVA

### Effect of switching cART on response inhibition in fMRI SST

In both studies, the main effects of activation patterns for both contrasts were in line with previous meta-analysis data on the SST task. In the NNRTI study, a reduction in BOLD activation in the STOP > GO contrast was observed after switching efavirenz to rilpivirine in the right IFG (pars opercularis), right insula, right prefrontal cortex, right posterior parietal cortex, right lateral occipital gyrus and left primary, and associative auditory cortex and left primary motor cortex, but these results did not survive after correction for multiple comparisons (FWE). This was also the case for the inverse contrast rilpivirine to efavirenz, where BOLD activation was observed in the right paracingulate cortex, right IFG (pars orbitalis and pars opercularis), bilateral frontal eye fields, right middle frontal gyrus, right pre-SMA, left posterior parietal cortex, right lateral occipital gyrus and right angular gyrus. The reverse contrast, GO > STOP, did not show any significant result. In the INSTI study, the analysis did not show any area of increased or decreased connectivity between time points in GO > STOP or STOP > GO contrasts.

## Discussion

Using a combined fMRI and cognitive behavioural approach, we were able to identify a dysfunction of the attentional network and executive functions that were restored by drug switching. From a neuropsychological perspective, in the NNRTI study, despite participants being asymptomatic, a significant improvement in sleep quality (measured by the PSQI) and anxiety symptoms (HADS) was observed, while in the INSTI study, we found no changes in any of the PROMs, which is in line with previous data (Mora-Peris et al. 2019), suggesting the observed changes are not just an artefact of repeated measurement. Moreover, these results are in line with previous data reporting that switching from efavirenz containing to efavirenz-free regimens has a beneficial impact on sleep quality and anxiety, as well as on executive function and attention (Vera et al. 2019; Walker and Brown 2018; Knoch et al. 2006). Interestingly, executive function was also found to be positively affected by drug switching in our study, measured by an improvement on the Groton Maze Learning Test and moreover on the SST task, which has become a standard measure of response inhibition (Logan and Cowan 1984). Data on SST performance in PWH are scarce, though a recent study reported that drug-naïve PWH showed comparable SSRTs with respect to healthy controls but longer RTs (Du Plessis et al. 2015). In the NNRTI study, we found that SSRTs were longer in participants on efavirenz before switching to rilpivirine, suggesting a detrimental effect caused by efavirenz with a loss of correct response inhibition, which might be restored by switching to rilpivirine. Moreover, we observed that the change in SSRTs between the two conditions was directly correlated with the duration of efavirenz treatment. The longer the patients were on efavirenz, the longer the SSRTs were and the worst the efficacy of response inhibition was. We postulate that this might reflect a cumulative effect of efavirenz treatment on the ability to correctly inhibit responses.

Functional connectivity has been extensively used in HIV clinical research settings (Ernst et al. 2002; Chang et al. 2001; Schweinsburg et al. 2012). Nevertheless, studies on drug-induced changes on resting-state networks in HIV populations are limited. If cART as a whole is taken into consideration, drug-naïve PWH show a lower functional connectivity in the DMN compared with healthy controls that could be restored after 12 weeks of cART (Zhuang et al. 2017). Interestingly, other experimental techniques such as MEG report that even PWH on effective cART show a disruption of the DMN compared with healthy controls (Becker et al. 2013), which could be explained by either the beneficial effect of cART being limited in restoring the network dysfunction produced by HIV infection or cART exerting both positive and negative effects on this RSN. Besides the DMN, increasing observations point towards an important role of the DAN in HIV-associated cognitive disorders (Ernst et al. 2002). The DAN is responsible for covert spatial attention, saccade planning and visual working memory (Vossel et al. 2014). Several fMRI studies have shown a greater load-dependent increase in activation in brain regions related to visual attention tasks (especially in the prefrontal and posterior parietal cortex) in PWH compared with healthy controls (Chang et al. 2008; Ernst et al. 2009), suggesting an increased brain metabolism due to an enhanced neuronal activity that could compensate for the underlying injury to the neural substrate caused by HIV infection. The driver of this attentional overload in PWH is largely unknown, and our study is one of the first to specifically assess the issue of the changes exerted by different cART regimens on the DAN.

In the NNRTI study, in PWH switching to an efavirenz-free cART, we observed improved attention through changes in the DAN. Specifically, our data indicate that the right superior parietal lobule (SPL) is the brain area which shows the highest vulnerability to efavirenz-induced damage. The role of the SPL in visuospatial attention has been previously reported by independent groups and different techniques (Fink et al. 2000; Fink et al. 2001; Wu et al. 2016; Vallar et al. 2014) and shows clear right lateralization, with the right posterior SPL showing stronger anatomical connections with the ipsilateral middle frontal gyrus (MFG) and inferior frontal gyrus (IFG), and with the contralateral posterior parietal cortex (PPC) than the left posterior SPL (Wu et al. 2016), accounting for the right predominance of visuospatial attention control. Interestingly, recent fMRI and MEG studies showed that the right SPL expresses the highest attentional load effect both in healthy controls and PWH under cART (Chang et al. 2008) and correlates with cognitive impairment in PWH (Becker et al. 2013). Therefore, the clear right lateralization of our finding is not surprising, though a bigger sample would be required to confirm its specificity. It is possible that efavirenz may cause a dysfunction of the DAN that is ameliorated, at least partially, by switching to rilpivirine. Moreover, since the DAN is activated during top-down feature-based attention, this would well reflect the improvements in the Groton Maze Learning Test, Chase Test and One Back Test. Therefore, while our results are in line with previous data on the role of the DAN dysfunction in PWH, we were able to dissect the differential impact that specific drugs can exert on this RSN.

The involvement of the VISAS, which is responsible for higher-level visual processing such as viewing complex stimuli and discrimination of locations in space, is not a surprising finding, given the fact that it constitutes one of the main projections to the DAN. Indeed, in the INSTI study, the finding of an enhanced connectivity in the left regions of the DAN and the right VISAS which are functionally highly correlated to the right regions of the DAN may suggest a compensatory mechanism due to an underlying damage of visuospatial processing and perceptual abilities. Sensory-motor function has been traditionally regarded as significantly impaired in PWH, especially in more severe cases and in the pre-cART era (Ances and Clifford 2008). However, it is still largely unknown the impact of different cART regimens on this network and further studies are needed in order to validate the relevance of this result.

There are limitations to our work. First, there is no HIV-negative control arm, and therefore, a comparison between subjects switching NNRTI and INSTI and healthy controls would have strengthened our interpretation of the comparisons found. However, the INSTI group findings, where no change was observed, would suggest that the NNRTI findings were the result of the switch form from efavirenz to rilpivirine rather than a repeated measures effect. Although De Plessis et al. (Du Plessis et al. 2015) showed comparable SSRTs between healthy controls and drug-naïve PWH, no study is currently available on SSRT differences between healthy controls and people PWH on efavirenz or rilpivirine containing cART. Secondly, the PROMs are self-reported questionnaires, so we cannot fully exclude a response shift bias, as is an intrinsic limitation of all self-reported questionnaires. Thirdly, these results should be considered exploratory, given the small sample size may limit our power of observation. As an example, we have applied fMRI power (Mumford 2012) to our current results in the NNRTI study to estimate the sample required to detect a difference in the insula for the contrast STOP > GO. We estimated that for 80% power, a group of 30 participants should be sufficient. Moreover, it is known that physiological noise such as cardiac and respiratory signals can bias fMRI results. Data required for sophisticated correction procedure such as physiological noise model (PNM) was not available for this study, and we based our denoising procedures on ICA-AROMA and visual assessment of noise components. Given that our areas of interest are far from the anatomical structures that are most susceptible to these artefacts (e.g. brainstem), we are confident that our data reflect a neural effect. Finally, we used 2 slightly different approaches for the ICAs of the 2 datasets: while in the INSTI study, we a priori set the number of components at 20; in the NNRTI, we let this number to be determined by the algorithm. Both approaches are valid and used within the neuroimaging community; in our case, the specific method was chosen to ensure the best fitting of our data to previously described RSNs. From a clinical perspective since attention disturbances, insomnia and anxiety are among the most common side effects of both efavirenz and dolutegravir and account for a high degree of therapy discontinuation (Apostolova et al. 2015; Hoffmann et al. 2017), we believe that the improvements in PSQI and HADS could play a major role in increasing medication adherence, while we argue that the change in functional connectivity of the DAN, VISAS and sensory-motor network could explain the reported lack of concentration and slower psychomotor function.

## Conclusion

In conclusion, evidence that fMRI changes are detectable in virologically suppressed, neurologically asymptomatic PWH on cART provides encouraging data on the potential use of these techniques as possible biomarker of CNS injury related to cART.
